# MBDDiff: an R package designed specifically for processing MBDcap-seq datasets

**DOI:** 10.1186/s12864-016-2794-z

**Published:** 2016-08-18

**Authors:** Yuanhang Liu, Desiree Wilson, Robin J. Leach, Yidong Chen

**Affiliations:** 1 Greehey Children’s Cancer Research Institute, University of Texas Health Science Center at San Antonio, San Antonio, TX USA; 2 Department of Cellular and Structure Biology, University of Texas Health Science Center at San Antonio, San Antonio, TX USA; 3 Department of Urology, University of Texas Health Science Center at San Antonio, San Antonio, TX USA; 4 Department of Epidemiology & Biostatistics, University of Texas Health Science Center at San Antonio, San Antonio, TX USA

**Keywords:** MBDCap-seq, DNA methylation, Differentially methylation, Differential methylated regions, MBDDiff, XBSeq

## Abstract

**Background:**

Since its initial discovery in 1975, DNA methylation has been intensively studied and shown to be involved in various biological processes, such as development, aging and tumor progression. Many experimental techniques have been developed to measure the level of DNA methylation. Methyl-CpG binding domain-based capture followed by high-throughput sequencing (MBDCap-seq) is a widely used method for characterizing DNA methylation patterns in a genome-wide manner. However, current methods for processing MBDCap-seq datasets does not take into account of the region-specific genomic characteristics that might have an impact on the measurements of the amount of methylated DNA (signal) and background fluctuation (noise). Thus, specific software needs to be developed for MBDCap-seq experiments.

**Results:**

A new differential methylation quantification algorithm for MBDCap-seq, MBDDiff, was implemented. To evaluate the performance of the MBDDiff algorithm, a set of simulated signal based on negative binomial and Poisson distribution with parameters estimated from real MBDCap-seq datasets accompanied with different background noises were generated, and then performed against a set of commonly used algorithms for MBDCap-seq data analysis in terms of area under the ROC curve (AUC), number of false discoveries and statistical power. In addition, we also demonstrated the effective of MBDDiff algorithm to a set of in-house prostate cancer samples, endometrial cancer samples published earlier, and a set of public-domain triple negative breast cancer samples to identify potential factors that contribute to cancer development and recurrence.

**Conclusions:**

In this paper we developed an algorithm, MBDDiff, designed specifically for datasets derived from MBDCap-seq. MBDDiff contains three modules: quality assessment of datasets and quantification of DNA methylation; determination of differential methylation of promoter regions; and visualization functionalities. Simulation results suggest that MBDDiff performs better compared to MEDIPS and DESeq in terms of AUC and the number of false discoveries at different levels of background noise. MBDDiff outperforms MEDIPS with increased backgrounds noise, but comparable performance when noise level is lower. By applying MBDDiff to several MBDCap-seq datasets, we were able to identify potential targets that contribute to the corresponding biological processes. Taken together, MBDDiff provides user an accurate differential methylation analysis for data generated by MBDCap-seq, especially under noisy conditions.

**Electronic supplementary material:**

The online version of this article (doi:10.1186/s12864-016-2794-z) contains supplementary material, which is available to authorized users.

## Background

The human genome is composed of billions of heritable non-static epigenetic arrangement of histone and DNA sequence that controls how genes are expressed [1] DNA methylation, along with some other covalent modifications of histone or DNA sequences, have regulatory control over gene expression. In 1975, two key publications have suggested that methylation of cytosine residues in the context of CpG dinucleotide could be an epigenetic marker of DNA sequences [2, 3]. A large majority of CpG islands of the vertebrate genome reside in or near the promoter regions [4]. Since then, DNA methylation has been intensively studied and, specifically, DNA methylation in promoter regions has been shown to be associated with cell development, tumor progression, and aging [5–7]

Through years of efforts, many experimental methods have been developed to assay the methylation status of CpG in a genome-wide manner. Currently, the golden standard of genome-wide profiling of DNA methylation is the whole genome bisulphite sequencing, which involves treatment of sodium bisulphite followed by high throughput sequencing (BS-seq) [8]. However, there are two major disadvantages for BS-seq. Firstly it requires genome-wide deep sequencing in order to precisely identify modification at base-pair level, which currently is not cost effective. Secondly, datasets generated by BS-seq might also give rise to alignment difficulties due to C/T modification. On the other hand, affinity-based method, such as methylated DNA immunoprecipitation followed by high throughput sequencing (MeDIP-seq) [9] and methyl-CpG binding domain-based capture followed by high throughput sequencing (MBDCap-seq) [8], are established as alternatives to BS-seq for genome-wide DNA methylation profiling which are more cost effective. It has been shown that MeDIP-seq is more sensitive to highly methylated, high-CpG densities regions and MBDCap-seq is more sensitive to highly methylated, moderate-CpG densities [10].

MBDCap-seq approach uses methyl-CpG binding domain of the MBD2 protein to capture double-stranded DNA, combined with subsequent high throughput sequencing, to systematically identify methylated regions in the genome. There are some unique characteristics of MBDCap-seq. DNA methylation profiling by MBDCap-seq is biased by underlying CpG properties of the genome, more precisely,, methylated regions with high GC contents are more likely to be eluted than regions with low GC contents. In terms of quantification of DNA methylation levels and the determination of differential methylation (DM), current methods used for testing of differential methylated regions (DMRs) generally do not take the sample specific background noise during MBD capture, which is caused by non-specific pull-down behavior of methyl-CpG binding domain, into consideration. Last but not least, up till now, there is no software that is designed specifically for processing large MBDCap-seq datasets. Previously, we developed an algorithm called BIMMER for testing genome-wide differential methylation, where we constructed a two-layer hidden Markov model (HMM) to model the differential methylation status [10]. However, because of the complexity of the algorithm and the nature of Expectation-Maximization (EM) solution, BIMMER is relatively slow in speed and is not suited for analyzing MBDCap datasets in large scale. Our aim for the study presented here is to provide an efficient computational pipeline specifically designed for identification of DM genes by using MBDCap-seq protocol.

## Methods

### Genome-wide MBDCap sequencing for prostate cancer patient samples

MBDCap-seq protocol was carried out to identify methylated regions across the genome for a set of prostate cancer samples as listed in Table 1. Total of 6 primary prostate tumors derived from patients that have different clinical outcome (3 Metastasized (METs) and 3 No Evidence of Disease (NEDs)) were processed and sequenced. Methylated fragments, bound to a methyl-CpG binding domain protein, were eluted for sequencing with the Illumina HiSeq 2000 sequencer with 50 bp single read (SR) sequencing module. Approximately 295 million sequence reads were generated and around 78 % reads were mapped to unique genome locations for all 6 samples. The MBDCap-seq analysis pipeline is:

**Table 1:** 6 prostate tumor samples profiled with MBDCap

Sample ID	Outcome	Gleason	Reads (millions)	Mapped reads (millions)
1	MET	7	51.57	40
2	MET	7	50.43	40.54
3	MET	10	53.43	41.83
4	NED	6	45.71	34.63
5	NED	7	47.05	27.03
6	NED		46.49	44.85

1. Apply FastQC to short read sequences to examine sequencing QC and other characteristics of sequence reads. Extract the file fastqc_data.txt for GC enrichment analysis;
2. Perform BWA aligner to align sequence reads to UCSC human genome build hg19 [11];
3. Remove sequence reads with equal and more than 2 bp mis-match and non-uniquely mapped to the genome;
4. Sort, convert and index BAM file for each sample;
5. Count number of reads in 100 bp bins tiling through entire genome by using BedCoverage/BedTools [12];
6. Count number of reads within 4kbp regions (+/- 2kbp around transcription start sites (TSS)) of each gene by using BedCoverage tool.

### Determination of GC enrichment

Normal human genome has GC content percentage roughly around 40 % (see UCSC genome statistics at http://genome.ucsc.edu/goldenPath/stats.html#hg18). The FastQC algorithm generates a GC counting statistic from all reads in fastqc_data.txt. If a sample contains portion of DNA that are enriched in GC content, we expect to see a shifted distribution, as illustrated in the Results section Fig. 4, to the right side of the normal genomic DNA GC distribution. Thus, by assuming a mixture model of 2 Gaussian distributions (gray-dashed line in Fig. 4), or *G* = *p*_1_*N*(*μ*_1_, *σ*_1_) + *p*_2_*N*(*μ*_2_, *σ*_2_), where *p*_1_ + *p*_2_ = 1, we can determine GC enrichment score,

**Fig. 1:**
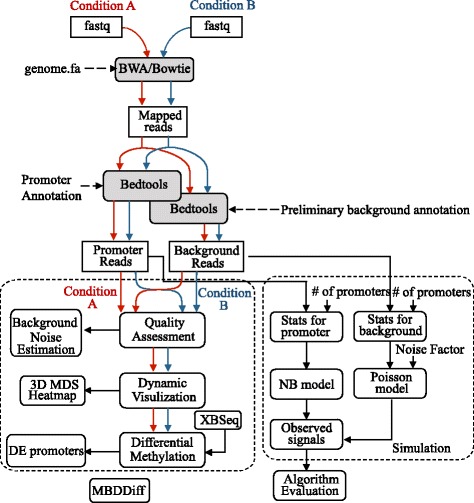
Workflow for MBDDiff and simulation procedure

**Fig. 2:**
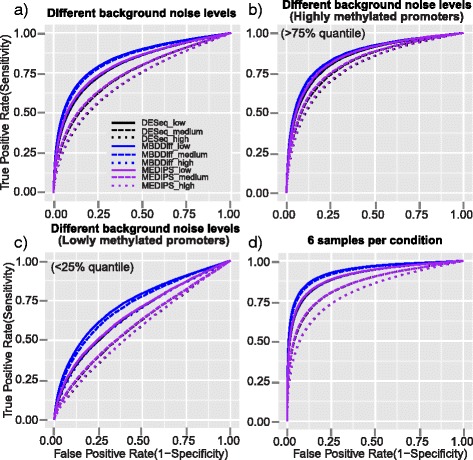
ROC curves of MBDDiff, MEDIPS and DESeq for simulated datasets in different scenarios. ROC curves for simulated MBDcap-seq datasets with low, intermediate or high level of background noise with 3 number of replicates in each group, 10 % of DM promoters with 2 fold of difference (**a**); Different levels of background noise but only for highly methylated promoters above 75 % quantile of methylation levels (**b**); Different levels of background noise but only for lowly methylated promoters below 25 % quantile of methylation levels (**c**); ROC curves for simulated MBDcap-seq datasets with low, intermediate or high level of background noise with 6 number of replicates in each group, 10 % of DM promoters with 2 fold of difference (**d**); Simulation was carried out 100 times and the average results is used

**Fig. 3:**
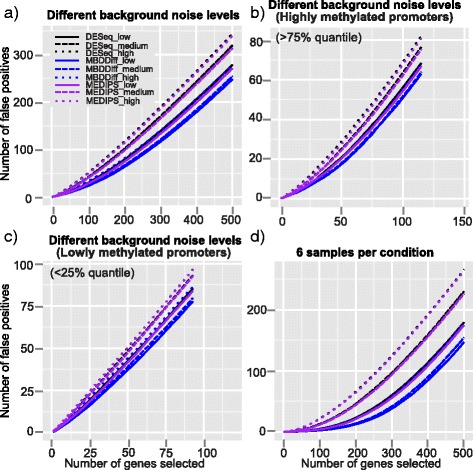
False discovery curves of MBDDiff, MEDIPS and DESeq for simulated datasets in different scenarios. False discovery curves for simulated MBDcap-seq datasets with low, intermediate or high level of background noise with 3 number of replicates in each group, 10 % of DM promoters with 2 fold of difference (**a**); Different levels of background noise but only for highly methylated promoters above 75 % quantile of methylation levels (**b**); Different levels of background noise but only for lowly methylated promoters below 25 % quantile of methylation levels (**c**); False discovery curves for simulated MBDcap-seq datasets with low, intermediate or high level of background noise with 6 number of replicates in each group, 10 % of DM promoters with 2 fold of difference (**d**); Simulation was carried out 100 times and the average results is used

**Fig. 4:**
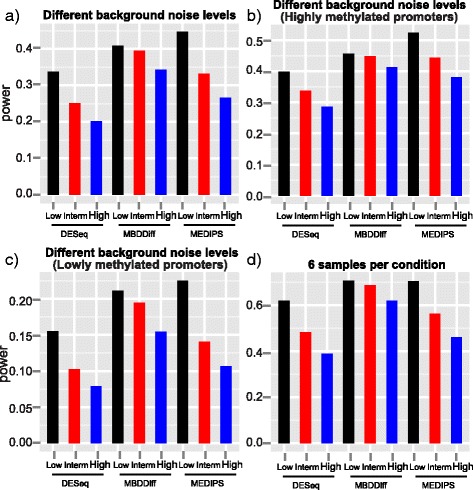
Statistical power of MBDDiff, MEDIPS and DESeq for simulated datasets in different scenarios. Bar plot of statistical power for simulated MBDcap-seq datasets with low, intermediate or high level of background noise with 3 number of replicates in each group, 10 % of DM promoters with 2 fold of difference (**a**); Different levels of background noise but only for highly methylated promoters above 75 % quantile of methylation levels (**b**); Different levels of background noise but only for lowly methylated promoters below 25 % quantile of methylation levels (**c**); Bar plot of statistical power for simulated MBDcap-seq datasets with low, intermediate or high level of background noise with 6 number of replicates in each group, 10 % of DM promoters with 2 fold of difference (**d**); Simulation was carried out 100 times and the average results is used

1 $$ ES={p}_2\left({\mu}_2-{\mu}_1\right)/\left({p}_1\times 20\right) $$

We initialized the model fitting with following parameters (for human genome), *μ*_1_ = 40, *μ*_2_ = 60, and *p*_1_ = *p*_2_ = 0.5. For our default setting (50 % reads are GC enriched), *ES* = 1.0. If only 20 % reads are enriched for GC content (*p*_2_/*p*_1_ = 20/80 = 1/4), we will have *ES* = 0.25, assuming other parameters stay the same. We select samples with *ES* > 0.2, otherwise, samples will be discarded without further data analysis.

### Construction of reference regions to measure background noise

Gene annotation (refFlat table) of human genome hg19 build was downloaded using UCSC table browser (http://genome.ucsc.edu/). Promoter regions were defined as regions ranging from 2 Kb upstream of TSS to 2Kb downstream of TSS. We only kept one TSS for transcripts with same TSS. In total, we constructed annotation for 33,178 promoters. To identify regions that potentially contribute to background noise, we built a 100 bp tiling window across the whole genome. We then used BEDTools to count the number of mapped reads within each tiling window of the 6 prostate cancer samples. In order to identify the regions for measuring background noise, we applied following procedures:

1. Filtering step: We exclude any 100 bp genome-wide tiling windows that reside in promoter regions, predicted CpG islands regions and any windows that contain ambiguous bases (gaps);
2. Construction Step: We then select preliminary background regions based on GC content as follows: for each promoter region, identify 80 100 bp windows nearby that has low in GC content (<40 %) and also relatively proximal to the corresponding TSS; and
3. Finalize background regions based on average transcript per million (TPM): for each promoter region, choose 40 out of 80 100 bp windows that are relatively low in *TPM* [13] as defined as $TPM = \frac{r_g\times {r}_l\times {10}^6}{f{l}_g\times {\displaystyle {\sum}_G}\frac{r_g\times {r}_l}{f{l}_g}},$ where *r*_*g*_ is the number of reads mapped to each 100 bp window, *r*_*l*_ is the read length, *fl*_*g*_ is fragment length, in our case, 100.

### Statistical framework and differential methylation testing of MBDDiff algorithm

The read count for promoter region of gene *i* can be decomposed into two components, true signal *S*_*i*_, which is directly associated with real methylation level, and background noise *B*_*i*_, which is attributed mainly to the random pull-down events from the wet-lab procedure of MBDCap-seq. Previously, we have developed an algorithm, XBSeq [14], for testing for differential expression for RNA-seq experiments. Here we applied similar statistical framework for MBDCap-seq experiments. Simply speaking, we assumed that the true signal (what we would like to estimate) *S*_*i*_ possesses a negative binomial distribution and background noise *B*_*i*_ follows a Poisson distribution. Then the observed signal (what we typically measured) *X*_*i*_ is a convolution of *S*_*i*_ and *B*_*i*_, which is governed by a Delaporte distribution [15].

$$ {X}_i={S}_i+{B}_i $$

2 $$ {S}_i\sim NB\left({r}_i,{p}_i\right) $$

$$ {B}_i\sim Poisson\left({\lambda}_i\right) $$

We further assumed that background noise *B*_*i*_ and true signal *S*_*i*_ are independent. By default, we applied a non-parametric method for parameter estimation. The Poisson parameter *λ*_*ι*_ for *B*_*i*_ can be estimated as:

3 $$ {\lambda}_i=\frac{1}{m}{\displaystyle \sum_{j=1}^m}{b}_{ij} $$

where *b*_*ij*_ denotes estimated background noise for promoter *i* across *m* replicates for each condition. Following the estimation of Poisson parameter, we will be able to infer mean *μ*_*Si*_ and standard deviation *σ*_*Si*_ for each promoter region:

4 $$ {\mu}_{S_i}=E\left({S}_i\right)=E\left({X}_i\right)-E\left({B}_i\right) $$

5 $$ {\sigma}_{S_i}^2={\sigma}_{X_i}^2+{\sigma}_{B_i}^2-2\rho {\sigma}_{X_i}{\sigma}_{B_i}, $$

Then the parameters for negative binomial distribution can be estimated by

6 $$ {r}_i={\mu}_{S_i}^2/\left({\sigma}_{S_i}^{\hbox{'}\ 2}-{\mu}_{S_i}\right) $$

7 $$ {p}_i={\mu}_{S_i}/{\sigma}_{S_i}^{\hbox{'}\ 2} $$

where ${\sigma}_{S_i}^{\hbox{'}}$ denotes adjusted variance for *S*_*i*_. This has proven to be useful when the sample size is small [16]. Details regarding non-parametric parameter estimation can be found in our previous publication of XBSeq [14].

When sample size is relatively large (>5), the maximum likelihood estimation (MLE) is applied to estimate parameters. The likelihood function is given by

8 $$ \begin{array}{c}L\left({\theta}_i\right)={\displaystyle \prod_{j=1}^m}p\left({X}_{ij}\Big|{\alpha}_i,\ {\beta}_i,{\lambda}_i\right)\cdot {\displaystyle \prod_{j=1}^m}p\left({B}_{ij}\Big|{\lambda}_i\right)\\ {} = {\displaystyle \prod_{j=1}^m}{\displaystyle \sum_{k=0}^{X_{ij}}}\frac{\Gamma \left({\alpha}_i+\mathrm{k}\right){\beta}_i^k{\lambda}_i^{X_{ij}-k}{e}^{-{\lambda}_i}}{\Gamma \left({\alpha}_i\right)k!{\left(1+{\beta}_i\right)}^{\left({\alpha}_i+k\right)}\left({X}_{ij}-k\right)!}\cdot {\displaystyle \prod_{j=1}^m}\frac{\lambda_i^{B_{ij}}{e}^{-{\lambda}_i}}{B_{ij}!}\end{array} $$

which has no closed form. Broyden–Fletcher–Goldfarb–Shanno (BFGS) algorithm is used to estimate the parameters by iterative updating. *α*_*i*_ and *β*_*i*_ are parameters for gamma portion of Delaporte distribution which are related to negative binomial parameters by:

9 $$ {r}_i={\alpha}_i $$

10 $$ {p}_i=1/\left({\beta}_i+1\right) $$

After successful estimation of all parameters, differential methylation testing of each promoter between two groups (with read count *x* and *y*) will be carried out by using moderated Fisher’s exact test:

11 $$ p=\frac{{\displaystyle {\sum}_{p\left(a,b\right)\le p\left(x,y\right)}}p\left(a,b\right)}{{\displaystyle {\sum}_{all}}p\left(a,b\right)} $$

where *a* and *b* are constrained by *a + b = x + y*

### Simulation

In order to evaluate the performance of our method, we generated a set of simulated datasets where we can control the differential methylation status of each promoter region. In this study, true signal *S* was simulated from a negative binomial distribution and background noise *B* was simulated from a Poisson distribution with parameters estimated from real MBDCap-seq datasets. We compared MBDDiff with MEDIPS [17], an R package designed for general purpose DNA fragments enrichment experiments, such as MeDIP-seq and MBDCap-seq for their ability to detect differntially methylated regions. We also compared MBDDiff with DESeq [16], an algorithm originally designed for differential expression analysis where the background noise is not considered for testing of expression difference between two conditions. We choose DESeq algorithm due to the fact that it has similar signal distribution assumption (Negative binomial) and differential test statistic (Fisher’s Exact test).

We followed a similar simulation procedure described in XBSeq. Basically, to estimate model parameters from a given MBDCap-seq dataset, 5000 promoters were randomly selected with replacement after discarding promoters with relatively low mapped reads or larger dispersion (top 10 %). The true signal *S* was simulated from a negative binomial distribution based on the mean and variance estimated from the 5000 promoters. 10 % or 30 % of the promoters were selected to be differentially methylated with enrichment fold change either 2 or 3. We simulated experiments with either 3 or 6 replicate samples per group to examine the potential effect of the number of replicates. To simulate background noise *B,* we first simulated read counts for the selected 100 bp windows. Then for each promoter region, background noise *B* was the summation of the read counts from all the corresponding 100 bp windows. We generated background noise in three different scenarios, with different level of dispersion, to examine the performance of our method in normal and noisy conditions. Background noise with different dispersion levels were simulated for each 100 bp window from a hybrid model:

12 $$ {B}_{inc}\sim M* Norm\left(\mu, \sigma \right) $$

where *μ* is from a Poisson distribution *μ* ~ *Poisson*(*λ* + *NF*). In our simulation, we set *M* = 10, *σ* = 3. The noise factor *NF* can be chosen from 0, 7, 20, each represents experiments with low background noise, intermediate background noise and high background noise. Simulations were repeated 100 times and statistical metrics were evaluated based on the average performance.

We evaluated different algorithms (MBDDiff, MEDIPS and DESeq) for their ability to discriminate between differentially methylated and non-differentially methylated promoters in terms of the following metrics: area under the ROC curve, number of false discoveries, statistical power, false discovery rate at pre-selected *p* value cutoff, distribution of *p* values under null model where there are no differentially methylated promoters. We also examined the performance of different methods separately for lowly and highly methylated promoters to see whether the performance is affected by methylated level of the promoter.

In order to investigate performance of different methods when the underlying model assumption can not be met. We simulated true signal from normal distribution with parameters estimated from a real MBDcap-seq dataset and background noise from either normal or uniform distribution to see whether the performance of different methods is affected or not. For instance, to simulate background noise from normal distribution. We also applied equation (12). The difference is that, the parameter *μ* is from a normal distribution with parameters estimated from a real MBDcap-seq dataset.

### Additional DNA methylation datasets for testing

In addition to our in-house prostate cancer samples, we also applied our method MBDDiff to previously published MBDCap-seq datasets where we compared endometrial cancer patient with either recurrent or non-recurrent outcome. 3 patients in each group were selected and processed with different methods to identify potential factors that contribute to endometrial cancer recurrence. Details about the experiments can be found in GSE26592 [18].

Similarly, we also selected and processed three tumor and normal samples from public domain dataset GSE58020, where MBDCap-seq were carried out to investigate DNA methylation profile for tripe negative breast cancers (TNBCs) [19].

### Comparison with other software for MBDCap-seq datasets

We also compared our algorithm with some other methods for MBDCap-seq datasets, including MEDIPS (1.20.0), DESeq (1.20.0). All these evaluations were carried out under R version 3.2.0 and Bioconductor version 3.1. Details regarding simulation procedure and workflow of MBDDiff is illustrated in Fig. 1.

## Results

### Implementation of MBDDiff

In order to use MBDDiff, we need to construct species specific background annotations that will be used to measure background noise for MBDCap-seq datasets. Several background annotation files have already been constructed and can be downloaded from github page (https://github.com/Liuy12/MBDDiff_files). Please follow instructions from MBDDiff package if you want to construct background annotation for your organism of choice. Currently MBDDiff uses BEDTools to count reads mapped to promoter and background regions. So users need to have BEDTools installed on their computer.

After counting reads mapped to promoter and background regions, MBDDiff then applies quality control procedure to examine the quality of MBDCap-seq datasets. Differential methylation testing will be carried out by XBSeq algorithm. In addition, MBDDiff also provides a set of visualizing tools for MBDCap data analysis.

### Discrimination between DM and non-DM promoters

In order to compare MBDDiff, MEDIPS and DESeq for processing MBDCap-seq datasets, we generated synthetic datasets when the differential methylation status and fold change of each promoter region can be controlled. We followed the simulation procedure described in the Methods section. Briefly, methylation levels from 5000 promoter regions and their corresponding background noise were simulated with model parameters estimated from a given prostate cancer MBDCap-seq dataset. Among 5000 promoter region simulated, 10 % or 30 % promoters were designated for enrichment fold-change at 2 or 3. Different background noise levels were simulated and all statistical metric calculation were reported as an average over 100 repeats.

We first compared the three methods for their ability to discriminate between differentially methylated and non-differentially methylated promoters in terms of the area under the Receiver Operating Characteristic (ROC) curve (AUC). As shown in Fig. 2 and Additional file 1: Table S1 and S2, 6 sample per group generally performed better than 3 samples per group in terms of AUC under various conditions. Overall, MBDDiff performs better with a higher AUC compared to MEDIPS and DESeq with different levels of background noise. For instance, under the condition of 3 samples per group, 10 % of differentially methylated promoters and 2-fold difference between two groups, MBDDiff achieved AUC 0.84 when background noise is relatively low, while the AUCs for MEDIPS and DESeq are 0.81 and 0.80 respectively. Even though all three methods have decreased AUC when background noise is increased (MBDDiff drop 0.04 to 0.80, MEDIPS dropped 0.1 to 0.71, and DESeq also dropped 0.1 to 0.70), MBDDiff is relatively resistant to higher background noise and performs much better even when the background noise is at very high level. We then examined the performance of the three methods separately for highly methylated (>75 %) and weakly methylated (<25 %) promoters. MBDDiff performs only slightly better compared to MEDIPS and DESeq for highly methylated promoters (Fig. 2b and Additional file 1: Figure S1a). However, for weakly methylated promoters, MBDDiff performs much better than MEDIPS and DESeq which indicates that background noise estimation procedure is essential for accurate DM detection under weakly methylation condition (Fig. 2c and Additional file 1: Figure S1b).

### Control of false discoveries

We then compared the performance of MBDDiff, MEDIPS and DESeq in terms of the false discoveries encountered among the top ranked differential methylated promoters based on *p* value. Overall, MBDDiff picked up the least number of false discoveries in various conditions (Fig. 3 and Additional file 1: Table S1 and S2). For example, under the condition of 3 samples per group, 10 % of differentially methylated promoters and 2-fold difference between two groups, MBDDiff identified 248 out of 500 number of false discoveries compared to MEDIPS (271) and DESeq (279) when the background noise has relatively low dispersion. When the background noise is increased, MBDDiff also picked up increased number of false discoveries (add 31 to 279), but is relatively resistant to the increase of background noise compared to MEDIPS (add 69 to 340) and DESeq (add 64 to 344). We also took a similar approach to examine the three methods separately for highly methylated (>75 %) and weakly methylated (<25 %) groups. The three methods picked up similar number of false discoveries for highly methylated promoters (Fig. 3b and Additional file 1: Figure S1c). However, for weakly methylated promoters, MBDDiff again performs the best with lowest number of false discoveries encountered (Fig. 3c and Additional file 1: Figure S1d). We also examined false discovery rate at pre-selected *p* value cutoff (*p* value = 0.05, Additional file 1: Figure S2c). MBDDiff has relatively low false discovery rate (around 0.4) compare to MEDIPS (around 0.5) and DESeq (around 0.5). Overall, MBDDiff is more robust against false discoveries compared to MEDIPS and DESeq especially for weakly methylated promoters even when the background noise is relatively high.

### Statistical power

Finally, we compared MBDDiff, MEDIPS and DESeq in terms of the statistical power at selected cutoff (*p* value = 0.05). As shown in Fig. 4 and Additional file 1: Table S1&2, MEDIPS performs the best when the background noise is relatively low. However, when we increased the dispersion of background noise, MBDDiff became the best method with the largest statistical power. For instance, under the condition of 3 samples per group, 10 % of differentially methylated promoters and 2-fold difference between two groups, MBDDiff achieved statistical power of 0.41 compared to MEDIPS (0.45) and DESeq (0.34) when the background noise is relatively low. However, when the background noise is higher, MBDDiff performs better with statistical power of 0.34 compared to MEDIPS (0.27) and DESeq (0.20). Similarly, we also compared statistical power of the three algorithms separately for highly methylated (>75 %) and weakly methylated (<25 %) promoters. For highly methylated group, MEDIPS performs slightly better than MBDDiff as shown in Fig. 4b and Additional file 1: Figure S1e. In contrast, for weakly methylated groups, MBDDiff performs much better than MEDIPS when the background noise is relatively high (Fig. 1f and 4c). Overall, MBDDiff remains one of the best method in terms of statistical power in various conditions and is more robust against the increase of background noise.

### Apply MBDDiff to prostate cancer datasets

**Fig. 5:**
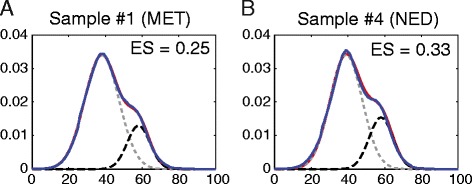
GC enrichment assessment. We perform GC enrichment test for all samples processed with MBDCap-seq protocol. **a** Enrichment score (ES) of 0.25 were detected for a MET sample. The dash lines are two Gaussian mixture models (light dash line for normal human genome GC distribution estimated from 50 bp short reads, and dark dash line for enriched 50 bp reads). ES score is evaluated by using Eq. 1. **b** ES of 0.33 for a NED sample. Both samples pass the threshold of > 0.20 requirement. In both (**a**) and (**b**), blue-line is the empirical density from the actual data, and red-line is the mixture model density. Both figure showed a tight estimation

**Fig. 6:**
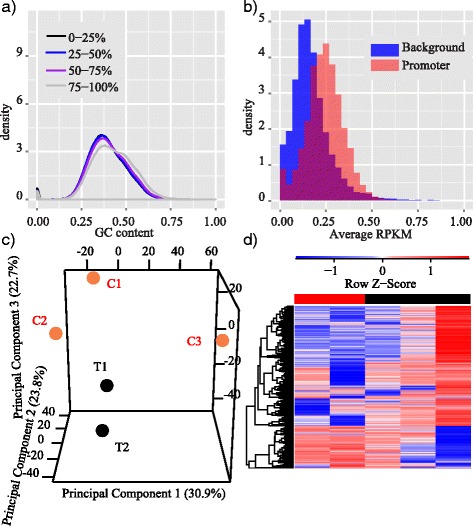
Apply MBDDiff to prostate cancer datasets. (**a**) Density plot of GC content grouped by different levels of methylation for all 100 bp windows across the whole genome of one example prostate MET sample; (**b**) Distribution of background noise and promoter counts; (**c**) 3D PCA plot of sample relation. C indicates NED patients and T indicates MET patients; (**d**) Heatmap of selected promoters for 5 prostate cancer samples

We recently carried out MBDCap-seq to examine whole genome DNA methylation profile in prostate tumors in order to identify potential factors that are involved in the development of prostate cancer after treatment. We excluded one patient sample because of a relative different Gleason score from other 5 samples. Then we applied MBDDiff to 5 samples in order to identify differentially methylated promoters between MET group and NED group. As shown in Fig. 6(a), there is a clear enrichment of GC content for highly methylated regions, which indicates the effectiveness in MBD2 capture procedure for our prostate samples. The enrichment of GC content was further assessed in Fig. 5, where 2 samples (one for MET and one for NED) were selected and their GC enrichment scores are 0.25 an 0.33, respectively. Figure 6(b) showed the distribution of background noise and promoter regions. We also examined the relationship between tumor samples. As we can observe from Fig. 6(c), patients with NED outcome are more dispersed than patients with MET outcome which might suggests a common mechanism for prostate cancer metastasis. After performing differential methylation algorithm, we identified 57 differentially methylated promoters with absolute log2 fold change greater than 1, *p*-value smaller than 0.001 and averaged methylation levels greater than 15 (read count unit). ANO7, a gene hyper-methylated in prostate cancer identified by our study, has been reported that it may act as a target gene for antibody-based immunotherapy [20]. F13A1, which is also identified as differentially methylated, is associated with bone metastasis in prostate cancer [21]. To conclude, by using MBDDiff, we were able to identify 57 promoters that were differentially methylated between MET and NED. This set of genes might be of helpful for future studies of prostate cancer metastasis.

### Apply MBDDiff to breast cancer datasets

**Fig. 7:**
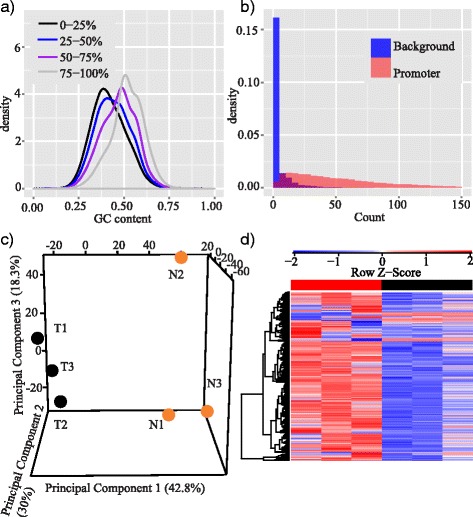
Functionalities for MBDDiff for processing TBNC datasets. **a** Density plot of GC content grouped by different levels of methylation for all 100 bp windows across the whole genome of one example TNBC sample; (**b**) Distribution of background noise and promoter counts; (**c**) 3D PCA plot of sample relation, T indicates tumor samples, N indicates normal samples; (**d**) Heatmap of selected promoters for 6 TNBC samples

To demonstrate the functionalities of MBDDiff, we also applied MBDDiff to a public dataset derived from TNBCs (GSE58020). We selected and processed 3 samples in both tumor and normal conditions. Firstly, MBDDiff assessed the quality of the samples by examining the GC enrichment for all 100 bp windows with different levels of methylation. As shown in Fig. 7(a), there is a clear enrichment of GC content for regions with higher mapped reads compared to ones with lower mapped reads which potentially contribute to background noise. After quality assessment, MBDDiff continued to estimate the context-specific background noise for each sample based on the preliminary background annotation. As shown in Fig. 7(b), background noise clearly coincides with the left hump of promoter mapped reads, indicating that the background noise we estimated indeed reflects the nature of mixture model for our observation. MBDDiff also provides some visualization functionalities including 3D principal component analysis (PCA) to examine sample relationship based on DNA methylation levels of promoter regions, more versatile heatmap visualization of DNA methylation levels across samples, etc. Alternatively, you can also generate an html report that contains several dynamic visualizations. Details can be found at our github page: https://github.com/Liuy12/MBDDiff.

As illustrated in Fig. 7(c), there is a clear separation between TNBC tumor and normal samples. Figure 7(d) showed heatmap of methylation levels of selected promoters across all samples (around 1800). Finally, MBDDiff performed differential methylation statistical tests based on XBSeq. Very interestingly, the dispersion for promoters showed a ‘S’ shape curve caused by promoters with either very small or very large dispersions, which probably is due to the artifacts of methylation measure from the MBDCap-seq protocol (Additional file 1: Figure S3A). Differential methylated promoters are identified by selecting promoters with absolute log2 fold-change greater than 1, adjusted *p*-value less than 0.01 and averaged methylation levels greater than 30. Additional file 1: Figure S3B showed the MA plot after differential methylation tests. With this stringent selection criterion, 348 unique promoters with significant differential methylation were obtained (significantly more than what we obtained in prostate cancer data, since here we compared tumor vs normal samples, while for prostate application, we studied tumors with NED outcome vs tumors with MET outcome). Interestingly, by using DAVID [22], we found that the genes of these promoters are enriched in biological processes, including regulation of transcription (adjusted *p*-value < 0.01) and regulation of neuron differentiation (adjusted *p*-value < 0.01), which might indicate the involvement of neuronal stem cell regulators in TNBCs [23].

### Apply MBDDiff to endometrial cancer datasets

**Fig. 8:**
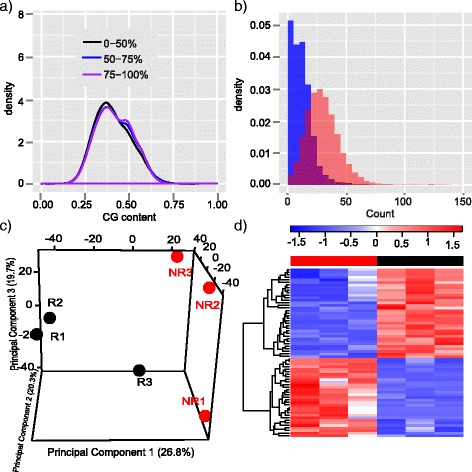
Apply MBDDiff to endometrial cancer datasets. **a** Density plot of GC content grouped by different levels of methylation for all 100 bp windows across the whole genome of one example endometrial sample with recurrence outcome; (**b**) Distribution of background noise and promoter counts for one example endometrial sample with recurrence outcome; (**c**) 3D PCA plot of sample relation, R indicates samples with recurrence outcome, NR indicates samples with non-recurrence outcome; (**d**) Heatmap of selected promoters for 6 samples

Finally, we applied MBDDiff to our previously published dataset where MBDCap-seq procedure was carried out to examine the global methylation patterns for a total of 232 primary samples in endometrial cohorts, breast cancer cohorts and breast cancer cell lines (GSE26592). For the purpose of testing our algorithm, we randomly selected three samples from endometrial cancer patients with either recurrent or non-recurrent outcome to identify potential factors that contribute to recurrence of endometrial cancer. After alignment procedure with bwa, the six samples were processed with MBDDiff. As shown in Fig. 8a, there is a clear enrichment of GC content for regions with relatively high methylation levels, which indicates that the dataset generated is of good quality. Figure 8b shows the distribution of background noise and true signal. Then MBDDiff assesses sample relationship based on methylation patterns of promoter regions. As shown in Fig. 8c, there is a clear separation between patients with recurrent and non-recurrent outcome. Figure 8d shows heatmap of promoter regions that are differentially methylated between the two groups. Finally, differential methylation test was carried out to identify potential factors that might cause recurrence of endometrial cancer. Totally we identified 66 differentially methylated promoters with fold change larger than 2, adjusted *p* value smaller than 0.1 and average mean methylation level bigger than 9. Compared to original paper we identified much less number of differentially methylated regions, the reason might be: 1) We only focus on differential methylation testing for promoter regions; 2) We only used 3 patient samples in each group. Among all the differential methylated genes, DKK1 has been shown to be a novel biomarker for endometrial carcinoma.

## Discussion

In order to compare MBDDiff, MEDIPS and DESeq, we carried out simulation procedure to generate datasets with different levels of background noise. Then we compared the three methods in terms of various statistical metrics. As we showed in the Results section, MBDDiff generally outperformed MEDIPS and DESeq in terms of AUC (Fig. 2) and number of false discoveries (Fig. 3). In terms of statistical power, MEDIPS performed slightly better than the other two methods when the background noise is relatively low. However, with higher background noise, MBDDiff has a better statistical power compared to MEDIPS and DESeq. Taken together, MBDDiff is more robust against higher background noise for accurately identifying differentially methylated promoters.

As designed in the simulation procedure, true signal was simulated from negative binomial distribution and background noise from Poisson distribution. While these are commonly consented models for data generated from NGS protocols, real data may deviate from NB and/or Poisson distributions. To investigate whether there is a potential bias with regards to the models we used for simulating true signal as well as background noise, we tested performance of the three methods with different models for true signal and background noise for simulation. Firstly, we simulated the true signal from a normal distribution and background noise from Poisson distribution (at normal level as estimated from the one selected prostate cancer dataset). As shown in Additional file 1: Figure S2a, irrespective of which models we used for simulation, MBDDiff performed better then the other two methods in terms of AUC (Additional file 1: Table S3). All these three methods tend to perform better when negative binomial is used for simulating true signal as we expected: the simulated data derived from models match the underlying models in these algorithms. Then we also tested the effect of using different models for simulating background noise (Additional file 1: Figure S2b). We used negative binomial model to simulate true signal but coupled with background noise derived from normal, uniform, or Poisson distribution. As shown in Fig S2b and Additional file 1: Table S4, MBDDiff outperformed MEDIPS and DESeq, regardless of which noise model you use for simulating background noise. In summary, MBDDiff is robust under different signal and background noise models. While for MEDIPS and DESeq, they work better when uniform and Poisson distributions (they virtually overlapping in Additional file 1: Figure S2b) rather than normal distribution for background noise. Potential bias can also be observed by examining the distribution of *p* values under a null hypothesis when no promoters are differentially methylated. As shown in Additional file 1: Figure S2d, *p* values generated by all three algorithms roughly follow the uniform distribution as we expected, with higher variation generated from MEDIPS.

We also benchmarked the three algorithms for experiments with different numbers of replicates. As shown in Additional file 1: Figure S4, DESeq consumes the most amount of time, followed by MBDDiff and MEDIPS. Higher computing cost for DESeq and MBDDiff with more replicate samples in each condition is mostly attributed to Fisher’s Exact test for assessing statistical significance.

## Conclusions

We developed an R package, MBDDiff, which aims specifically to process MBDCap-seq datasets. MBDDiff provide users the ability to assess quality of datasets, test for differential methylation of promoter regions and visualization functionalities.

## Abbreviations

AUC, area under the curve; BS-seq, bisulphite sequencing; DM, differential methylation; MBDCap-seq, methyl-CpG binding domain-based capture followed by high throughput sequencing; MeDIP-seq, methylated DNA immunoprecipitation followed by high throughput sequencing; MET, metastasis samples; NB, negative binomial (distribution, model); NED, no evidence of disease samples; ROC, receiver operating characteristic.

## Additional file

Additional file 1: Supplementary information. Supplementary figures and tables to provide additional analysis results. (PDF 1214 kb)

## References

[1] Inbar-Feigenberg M, Choufani S, Butcher DT, Roifman M, Weksberg R (2013). Basic concepts of epigenetics. Fertil Steril.

[2] Holliday R, Pugh JE (1975). DNA modification mechanisms and gene activity during development. Science.

[3] Riggs AD (1975). X inactivation, differentiation, and DNA methylation. Cytogenet Cell Genet.

[4] Deaton AM, Bird A (2011). CpG islands and the regulation of transcription. Genes Dev.

[5] Heyn H, Esteller M (2012). DNA methylation profiling in the clinic: applications and challenges. Nat Rev Genet.

[6] Gentilini D, Mari D, Castaldi D, Remondini D, Ogliari G, Ostan R, Bucci L, Sirchia SM, Tabano S, Cavagnini F (2013). Role of epigenetics in human aging and longevity: genome-wide DNA methylation profile in centenarians and centenarians’ offspring. Age.

[7] Robinson MD, Stirzaker C, Statham AL, Coolen MW, Song JZ, Nair SS, Strbenac D, Speed TP, Clark SJ (2010). Evaluation of affinity-based genome-wide DNA methylation data: effects of CpG density, amplification bias, and copy number variation. Genome Res.

[8] Taiwo O, Wilson GA, Morris T, Seisenberger S, Reik W, Pearce D, Beck S, Butcher LM (2012). Methylome analysis using MeDIP-seq with low DNA concentrations. Nat Protoc.

[9] Li N, Ye M, Li Y, Yan Z, Butcher LM, Sun J, Han X, Chen Q, Zhang X, Wang J (2010). Whole genome DNA methylation analysis based on high throughput sequencing technology. Methods.

[10] Mao Z, Ma C, Huang TH, Chen Y, Huang Y (2014). BIMMER: a novel algorithm for detecting differential DNA methylation regions from MBDCap-seq data. BMC Bioinformatics.

[11] Li H, Durbin R (2009). Fast and accurate short read alignment with Burrows-Wheeler transform. Bioinformatics.

[12] Quinlan AR (2014). BEDTools: the swiss-army tool for genome feature analysis. Curr Protoc Bioinformatics.

[13] Baccarelli A, Rienstra M, Benjamin EJ (2010). Cardiovascular epigenetics: basic concepts and results from animal and human studies. Circ Cardiovasc Genet.

[14] Chen HI, Liu Y, Zou Y, Lai Z, Sarkar D, Huang Y, Chen Y (2015). Differential expression analysis of RNA sequencing data by incorporating non-exonic mapped reads. BMC Genomics.

[15] Johnson NL, Kemp AW, Kotz S (2005). Univariate discrete distributions.

[16] Anders S, Huber W (2010). Differential expression analysis for sequence count data. Genome Biol.

[17] Lienhard M, Grimm C, Morkel M, Herwig R, Chavez L (2014). MEDIPS: genome-wide differential coverage analysis of sequencing data derived from DNA enrichment experiments. Bioinformatics.

[18] Gu F, Doderer MS, Huang YW, Roa JC, Goodfellow PJ, Kizer EL, Huang TH, Chen Y (2013). CMS: a web-based system for visualization and analysis of genome-wide methylation data of human cancers. PLoS One.

[19] Stirzaker C, Zotenko E, Song JZ, Qu W, Nair SS, Locke WJ, Stone A, Armstong NJ, Robinson MD, Dobrovic A (2015). Methylome sequencing in triple-negative breast cancer reveals distinct methylation clusters with prognostic value. Nat Commun.

[20] Bera TK, Das S, Maeda H, Beers R, Wolfgang CD, Kumar V, Hahn Y, Lee B, Pastan I (2004). NGEP, a gene encoding a membrane protein detected only in prostate cancer and normal prostate. Proc Natl Acad Sci U S A.

[21] Morrissey C, True LD, Roudier MP, Coleman IM, Hawley S, Nelson PS, Coleman R, Wang YC, Corey E, Lange PH (2008). Differential expression of angiogenesis associated genes in prostate cancer bone, liver and lymph node metastases. Clin Exp Metastasis.

[22] da Huang W, Sherman BT, Lempicki RA (2009). Systematic and integrative analysis of large gene lists using DAVID bioinformatics resources. Nat Protoc.

[23] Cimino-Mathews A, Subhawong AP, Elwood H, Warzecha HN, Sharma R, Park BH, Taube JM, Illei PB, Argani P (2013). Neural crest transcription factor Sox10 is preferentially expressed in triple-negative and metaplastic breast carcinomas. Hum Pathol.

