# Supplementary material for: MBDDiff: an R package designed specifically for processing MBDcap-seq datasets
Source: BMC Genomics. 2016 Aug 18;17(Suppl 4):432. doi: 10.1186/s12864-016-2794-z (PMC5001203; doi:10.1186/s12864-016-2794-z)
Supplement: Additional file 1: — Supplementary information. Supplementary figures and tables to provide additional analysis results. (PDF 1214 kb) [file 12864_2016_2794_MOESM1_ESM.pdf]

Model-based and context-specific background correction and differential methylation testing for MBDCap-seq

Yuanhang Liu[1,2], Desiree Wilson[2], Robin J. Leach[2,3], Yidong Chen[1,4,§]

Supplementary Figures

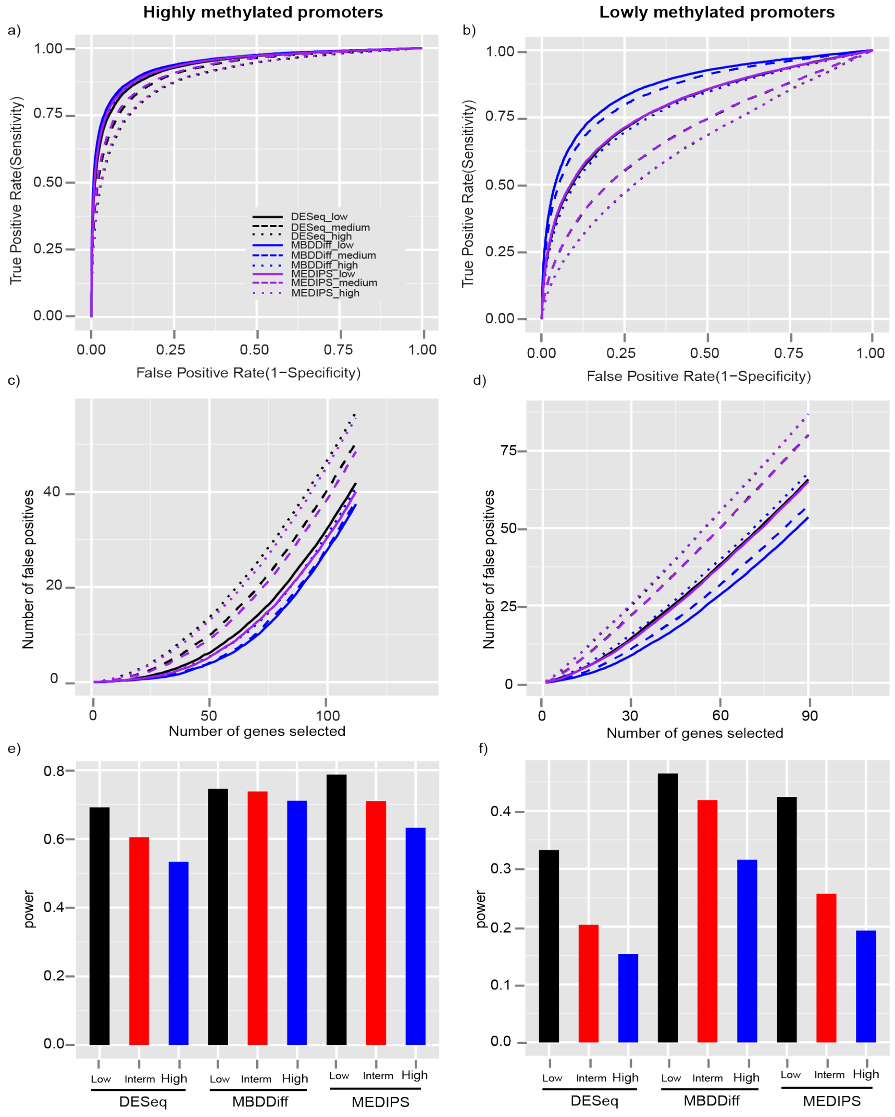

Figure S1. ROC, false discovery curve, power bar plot with highly methylated promoters (>75% quantile) and lowly methylated promoters (<25% quantile) with 6 number of replicates in each condition. Simulation was carried out 100 times and average values were used.

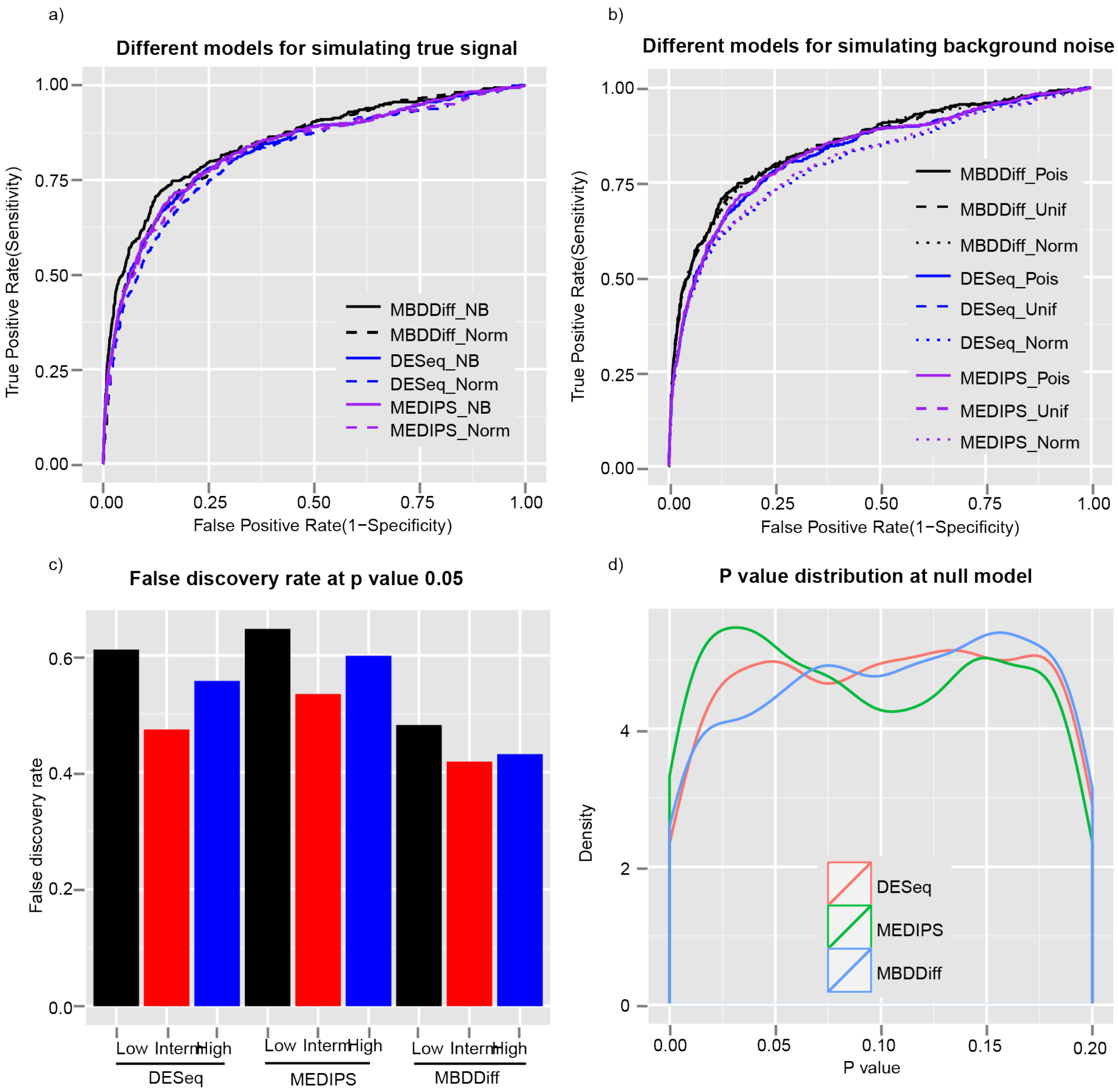

Figure S2. (a) ROC curves regarding simulation of true signal based on different statistical distributions, including normal and negative binomial distribution (b) ROC curves regarding simulation of background noise based on different statistical distributions, including Poisson, uniform and normal distribution. (c) False discovery rate at preset threshold with *p* value equals to 0.05 (d) The p value distributions under null model distribution (without differentially methylated promoters).

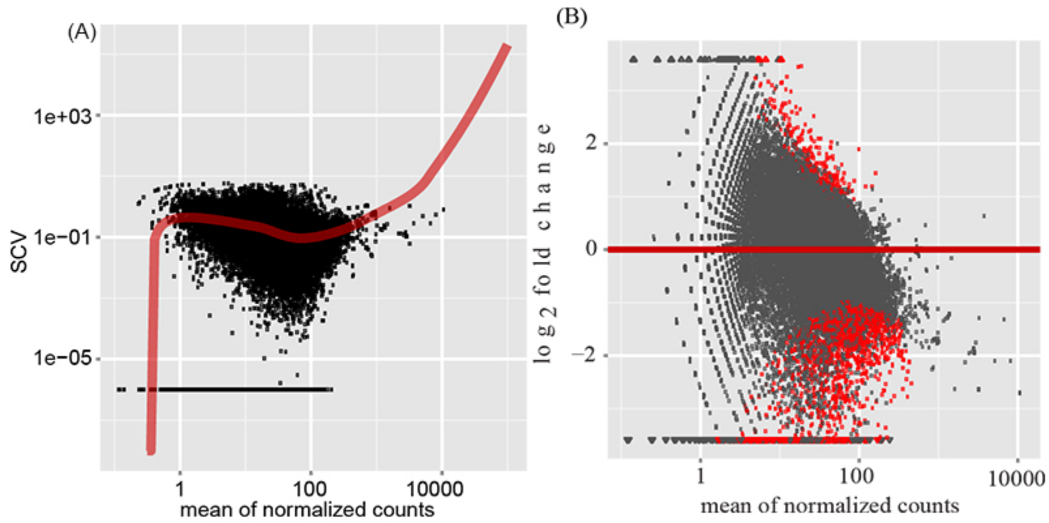

Figure S3 **Differential methylation analysis of TNBCs.** (A) Plot of dispersion vesus mean of normalized counts; (B) Log2 fold change vesus mean of normalized counts, red dots indicate differentially methylated promoters with *p*-value smaller than 0.01, absolute log2 fold change larger than 1.

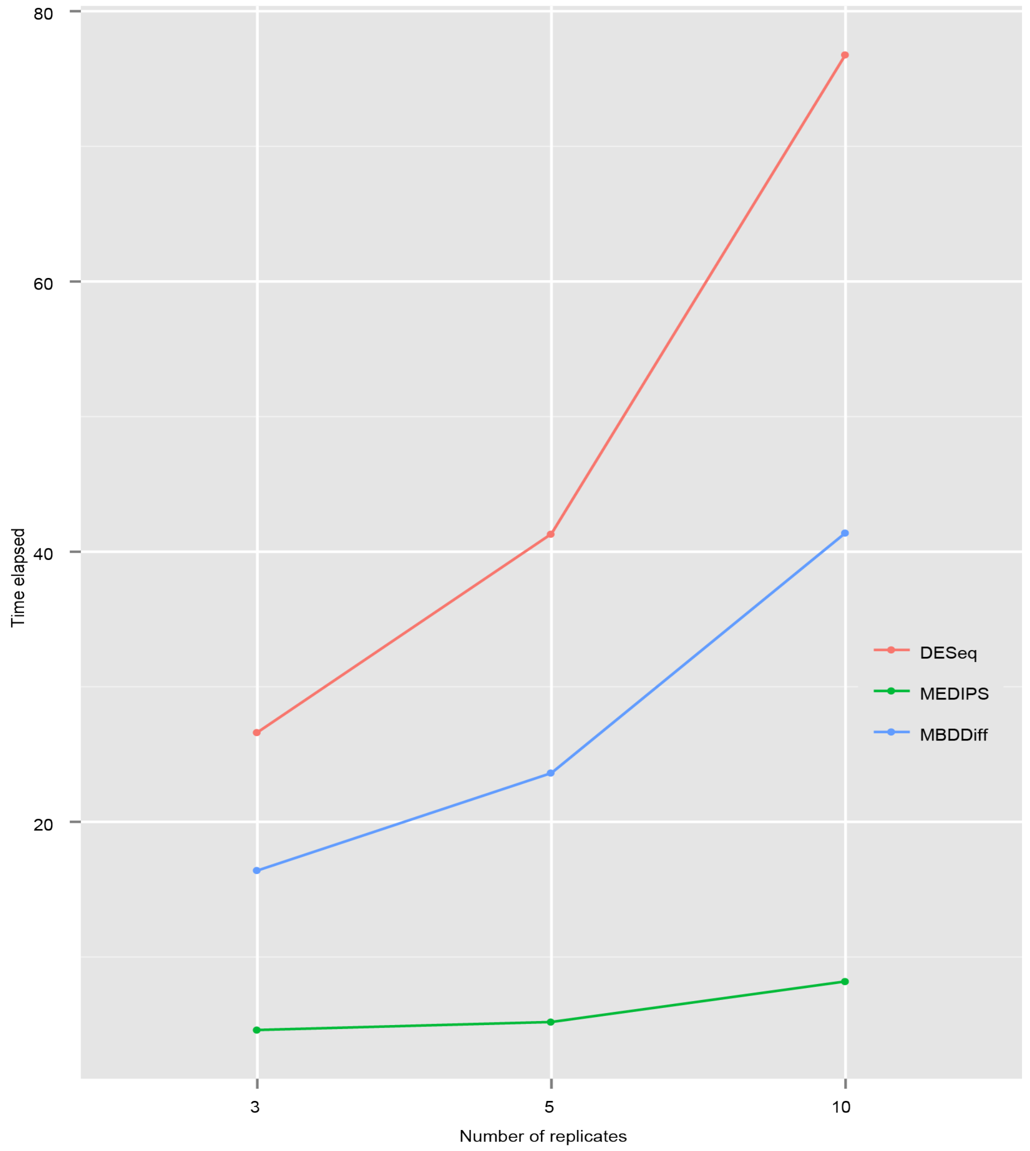

Figure S4. Execution time of different statistical methods with 3, 5, and 10 replicates in 2 conditions with relatively low background noise with three number of replicates (test vs control). The comparison was performed on red hat enterprise linux server release 6.3 with 80 CPUs of 1994.866 MHz.

Supplementary tables

		DESeq				MBDDiff				MEDIPS		
# of samples/group		Low	Interm	High		Low	Interm	High		Low	Interm	High
3 replicates 10% DMP	AUC	0.80	0.74	0.70		0.84	0.84	0.80		0.81	0.75	0.71
	# of FD	279	320	344		248	255	279		271	314	340
	Power	0.34	0.25	0.20		0.41	0.40	0.34		0.45	0.33	0.27
3 replicates 30% DMP	AUC	0.81	0.74	0.70		0.84	0.84	0.80		0.81	0.75	0.71
	# of FD	554	656	727		479	494	551		538	645	719
	Power	0.34	0.25	0.20		0.41	0.39	0.34		0.45	0.33	0.27
6 replicates 10% DMP	AUC	0.91	0.85	0.81		0.94	0.93	0.91		0.91	0.85	0.81
	# of FD	179	230	266		147	154	179		175	226	264
	Power	0.62	0.48	0.39		0.71	0.69	0.62		0.71	0.56	0.46
6 replicates 30% DMP	AUC	0.91	0.85	0.81		0.94	0.93	0.91		0.91	0.85	0.81
	# of FD	346	455	541		280	294	348		338	451	538
	Power	0.61	0.48	0.38		0.70	0.68	0.62		0.70	0.56	0.46

Table1. Values of AUC, number of false discoveries, statistical power for DESeq, MBDDiff and MEDIPS in various conditions with fold change equals to 2, 3 or 6 number of replicates per test group, 10% or 30% number of DM promoters

		DESeq				MBDDiff				MEDIPS		
# of samples/group		Low	Interm	High		Low	Interm	High		Low	Interm	High
3 replicates	AUC	0.92	0.88	0.83		0.95	0.95	0.92		0.93	0.88	0.84
10% DMP	# of FD	166	214	247		134	141	164		149	200	236
	Power	0.65	0.52	0.43		0.74	0.72	0.66		0.77	0.63	0.53
3 replicates	AUC	0.92	0.88	0.84		0.95	0.95	0.92		0.93	0.88	0.84
30% DMP	# of FD	310	411	491		242	256	309		282	390	473
	Power	0.64	0.51	0.43		0.73	0.72	0.66		0.77	0.63	0.53
6 replicates	AUC	0.98	0.95	0.92		0.99	0.99	0.98		0.98	0.95	0.92
10% DMP	# of FD	82	119	151		59	64	82		73	113	148
	Power	0.88	0.79	0.71		0.93	0.92	0.88		0.93	0.84	0.76
6 replicates	AUC	0.98	0.95	0.92		0.99	0.99	0.98		0.99	0.95	0.92
30% DMP	# of FD	151	230	299		101	113	150		139	226	296
	Power	0.88	0.78	0.70		0.93	0.92	0.88		0.92	0.84	0.76

Table2. Values of AUC, number of false discoveries, statistical power for DESeq, MBDDiff and MEDIPS in various conditions with fold change equals to 3, 3 or 6 number of replicates per test group, 10% or 30% number of DM promoters

	MBDDiff	MEDIPS	DESeq
Negative Binomial	0.85	0.83	0.83
Normal	0.84	0.81	0.82

Table3. Values of AUC, for simulation of true signal based on different models

	MBDDiff	MEDIPS	DESeq
Poisson	0.85	0.83	0.83
Normal	0.85	0.83	0.83
Uniform	0.85	0.80	0.81

Table4. Values of AUC, for simulation of background noise based on different models
